# Effectiveness of the Relative Strength Index Signals in Timing the Cryptocurrency Market

**DOI:** 10.3390/s23031664

**Published:** 2023-02-02

**Authors:** Marek Zatwarnicki, Krzysztof Zatwarnicki, Piotr Stolarski

**Affiliations:** 1 Department of Information Systems, Poznan University of Economics and Business, Niepodleglosci 10, 61-875 Poznan, Poland; 2 Department of Computer Science, Opole University of Technology, Proszkowska 76, 45-758 Opole, Poland

**Keywords:** decision-making system, trading indicator, RSI, technical analysis, cryptocurrency, divergence

## Abstract

In 2020 and 2021, the cryptocurrency market attracted millions of new traders and investors. Lack of regulation, high liquidity, and modern exchanges significantly lowered the entry threshold for new market participants. In 2021, over 5 million Americans were regularly involved in cryptocurrency trading. At that time, the interest in market indicators and trading strategies remained low, leading to the conclusion that most investors did not use decision-support indicators. The correct and backtested use of technical analysis signals can give the trader a significant advantage over most market participants. This work introduces an algorithmic approach to examining the effectiveness of the signals generated by one of the most popular market indicators, the Relative Strength Index (RSI). A model corresponding to an actual cryptocurrency exchange was used to backtest the strategies. The results show that the RSI as a momentum indicator in the cryptocurrency market involves high risk. Using alternative RSI applications can allow traders to gain an advantage in the cryptocurrency market. Comparing the results with the traditional buy and hold strategy shows the credible potential of the indicated method and the usage of signals generated by the technical analysis indicators.

## 1. Introduction

According to the Statista report from 2020, in the United States alone about 140 million American adults own stocks or other market investments [1]. In comparison, only 13.9 million people worldwide are short-term investors or traders in traditional markets [2,3]. The statistics look very different in the cryptocurrency market, which is a new, rapidly growing market sector that allows for speculation and investment in assets [4]. The profile of the average cryptocurrency owner differs significantly from that of the stock owner. According to the data collected by Gemini in the report “2021 State of Crypto in the US” [5], 58% of cryptocurrency owners say that they bought, sold, or traded cryptocurrency within the last three months. Thirty-six percent of the owners actively trade their assets to increase profit, which is over 5 million moderately active traders in the United States alone [5].

In 2021, twelve years after its creation and four years after the first broader wave of interest, Bitcoin again attracted the general public’s attention. According to Gemini, 95% of Americans have heard about Bitcoin, and 38% have heard about the second largest cryptocurrency, Ethereum. Currently, 14% of American adults own a fragment of at least one fractional unit of cryptocurrency, and 63% are interested in such an investment in the future [5].

**Figure 1:**
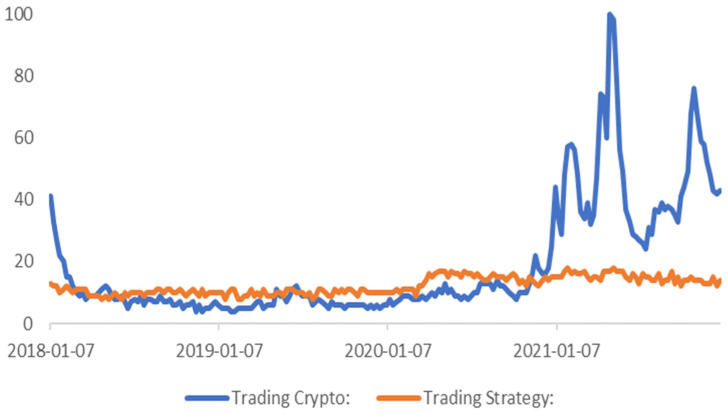
Google trends phrase search results for crypto trading and trading strategy in the period under the study.

Google trends statistics show (Figure 1) that despite the significant increase in interest in crypto trading, at the same time the interest in the market analysis and technical analysis (TA) indicators grew disproportionately slowly. Therefore, it can be assumed that the awareness and knowledge of the new market participants remain low. Statistically, the proven solutions can give a well-educated investor an advantage over most market participants [6,7].

TA uses constant repeating patterns and relationships from past price data to predict future price movements. It allows us to determine the market trend, capital inflows, outflows, and resistances on which price increases or decreases may stop [7,8]. Many researchers are skeptical about the potential success of the strategies based on the range of possibilities offered by TA, which contradicts the weak form of the Efficient Market Hypothesis (EMH) [9]. The hypothesis states that stocks are always traded at fair value. Therefore, it is impossible to obtain above-average results using price data only. Despite many studies confirming the effectiveness of the strategies based on technical analysis, its moderate criticism may be accurate as it does not consider only one of the many factors driving the market [10].

Another approach to market analysis is fundamental analysis (FA), focusing on the economic and financial aspects of the instrument under study [11]. In the case of cryptocurrencies, a large part of the FA was taken over by on-chain analysis [12]. A large proportion of investors use TA to support FA. However, many studies show that treating them as equal but different market elements allows for the building of a compelling investment system [13,14].

In the case of cryptocurrencies, social attitudes and interests also play an essential role. As shown by Stolarski [15], the number of searches of a given cryptocurrency on the Web can help predict the prices of a given cryptocurrency. Another popular market sentiment indicator is the Fear and Greed Index, which measures investors’ sentiment in social media [16].

In this research, we focus exclusively on the review of the results obtained thanks to the strategies in the field of TA. However, the presented evaluation methods can also be applied to FA and on-chain analysis. In the study, we assume that the price movements of the cryptocurrency selected for research include the changes caused by the movements on the blockchain and the influence of macroeconomic or fundamental phenomena [17]. Due to the possibility of a price response to the events occurring around the clock, on any day of the year, such an assumption may be more accurate than it is in the traditional markets.

The main goal of this work is the evaluation of the effectiveness of the well-known strategy applications of the Relative Strength Index.

The model corresponding to the crypto exchange was created to carry out the considered strategies backtest. The initial wide range of strategies, time intervals, and cryptocurrencies is examined. The representative portfolio is created using statistical methods. The in-detail experiments are conducted, and the results are confronted with popular opinion and the author’s recommendations regarding the usage of Relative Strength Index (RSI). The conclusions are made, and the profitable automated trading system is built based on the results.

The presented approach allows investors to potentially increase profit while significantly reducing potential capital downdraws in future investments. The comparison of the results with the traditional buy and hold strategy shows the credible potential of the indicated methodology and the usage of technical analysis indicators. Research has been conducted on the cryptocurrency market usage purpose, but the initial experiments show the potential of this approach in traditional markets.

The rest of the article is composed as follows. Section 2 presents the related work with a description of the previously selected works constituting the basis for a new solution. Section 3 contains the assumptions adopted during the research, the portfolio’s construction, and the characteristics of the market under study. In Section 4, the research results and a discussion of the results are presented. Section 5 summarizes the article.

## 2. Related Work and Motivation

In the past, many studies were conducted confirming the legitimacy of the use of TA in traditional markets. Such research was conducted, for example, by Kuang et al. and Jegadeesh [18,19]. Many researchers, including Allen and Taylor, indicate the irrationality of using TA [17]. For this reason, in recent years, the research in this field has been conducted more and more often only on emerging markets [20,21], confirming that, under the right conditions, it is possible to achieve above average results by using TA. The recent studies also show the potential of using TA in fully developed economies [22]. Recently, the popular approach has been to pre-act prices using neural networks and methods traditionally used in predictions and simulations, combining forecasting and TA [23,24,25]. The authors note that combining both methods can be successful and will be the subject of further research by the authors. However, the following work focuses on the classical application of technical analysis, without the use of learning systems.

Cryptocurrency exchanges such as Binance allow users to trade with a minimum commission of only a 0.1% value of the transaction, a high leverage of a max of 125× on high volumetric assets, trade every day of the year, trade p2p, and trade directly between cryptocurrency pairs without the need to previously exchange to fiat-based stablecoins [26,27]. Modern API (application programming interface), connected directly to a specific stock exchange without the need for a traditional broker to participate in the process, allows for a relatively easy connection of proprietary algorithms that automate trading [28,29]. Such a vast pool of new opportunities for people interested in the market and algorithmics allows for high passive income with the right approach.

All the above features that characterize the cryptocurrency market translate into high and rapid price volatility and high liquidity. This makes the market ineffective, and it shares many features with emerging markets. As it is for emerging markets, TA in the cryptocurrency market is likely to work better than in the more mature markets [30].

Using strategies based on the most known algorithms with the right approach can bring significant profits and gain an advantage over other less experienced market participants [31,32]. It also allows for a generation of buy and sell signals without manual analysis or for the automated buying and selling with the help of an automated trading system. Similar studies were conducted for traditional markets, but despite the clearly positive results, the awareness of these solutions among the general public remain low (Figure 1).

Most of the popular trading strategies relay on basic indicators such as the Moving Average Convergence Divergence (MACD), the Relative Strength Index (RSI), the Simple Moving Average (SMA), the Exponential Moving Average (EMA), or the newer trend following the indicator Supertrend [33,34,35]. Their basic applications, due to their popularity and the growth character of the market, may be exposed to market efficiency. It means that despite the results being close to the buy and hold strategy, they may not be the optimal solutions.

Extensive research on the effectiveness of one of the most popular technical analysis indicators, the Relative Strength Index (RSI), has shown that the use of the commonly known solutions in the traditional markets may not give the investor an advantage in the market. The study results lead to a thesis that the RSI and other cryptocurrency market indicators may prove equally ineffective. The use of alternative approaches to the RSI interpretation may, however, give the investor an advantage in the market [31].

In most studies, an exhaustive search is carried out to find the optimal applications of a chosen trading strategy [31,32]. It is a simple solution to find the optimum with a relatively low degree of the complexity of most strategies. Most studies only assess the optimal results exclusively through the prism of the portfolio’s final value obtained using selected parameters.

The motivation of the authors is to study the effectiveness of the RSI technical analysis indicator, one of the most popular indicators in the cryptocurrency market. The authors’ goal is to examine the popular strategies based on the RSI and to find new areas of application for the indicator. The authors propose a simple strategy as an extension of the most universally examined strategy in order to achieve better results. The authors examine the effectiveness of the new solution in the post-main research period and compare it to the popular strategies based on other indicators.

## 3. The Experiment Assumptions and Initial Studies

When researching trading strategies, it is necessary to make assumptions that allow the best possible representation of the market conditions a trader would face. The authors carefully analyzed the conditions prevailing in the cryptocurrency market in recent years, the performance of a wide range of cryptocurrencies, and the offer offered by cryptocurrency exchanges.

The following chapter describes the general assumptions that were adapted in order to conduct reliable research corresponding to actual events.

### 3.1. The Relative Strength Index Assumptions

This paper examines the historical effectiveness of one of the most popular technical analysis indicators, the Relative Strength Index (RSI). The RSI is an indicator that is available as one of the built-in features on most cryptocurrency exchanges [36].

The RSI is classified as a momentum oscillator. It measures the rate at which the price increases or decreases. The Relative Strength (RS) is calculated as the ratio of the higher to the lower close of the index. Indexes with higher RSI values usually experience more positive price changes. Similarly, a low RSI means more remarkable negative changes.

The RSI is most commonly used on a daily timeframe with a 14-day data window. It is measured on a scale between 0 and 100, where overbought is labeled as above 70, and oversold is labeled as below 30. Different values can be used in different interpretations. It can be used with other strategies to signal or confirm a decision [37].

Contrary to most indicators, this is a leading indicator used not to determine the market’s trend but to indicate in advance the potential trend reversals. The indicators determining the current market trend are lagging indicators [38].

The Relative Strength Index was developed and published in 1978 by J. Welles Wilder in his first book, *New Concepts in Technical Trading Systems*. It was also published in *Commodities* magazine (Futures) in June 1978. The idea caught on well, and the indicator quickly became one of the most popular technical analysis indicators and has remained so to this day [37].

The formula for the RSI used in the research was presented as follows [37]:

*RSI* = *Cumulated up movement of prices over the previous t periods/(Cumulated up movement of prices over the previous t periods* + *cumulative absolute downward movement over the previous t periods)* (1)

where *t* is the number of candles.

The data source used to calculate the RSI in the experiments was the close price of the previous Japanese candle. It is the most straightforward and commonly used data source in technical analysis. In this paper, we do not examine the results that can be obtained with other data sources.

**Figure 2:**
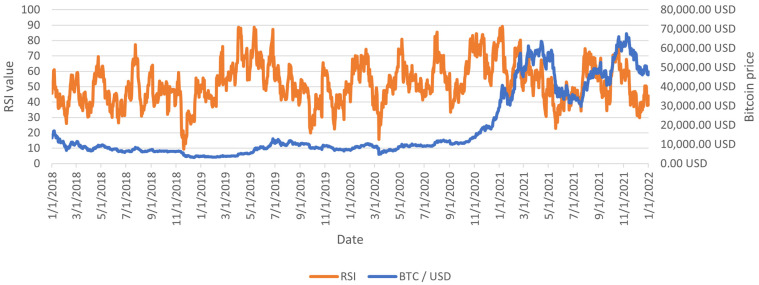
The Relative Strength Index calculated with close as input for Bitcoin on 1D interval.

There are infinite possible lengths of the RSI indicators. However, for the experiment, there was a basic factor length of 14 candles. This value is the most commonly used length, proposed by the inventor Wilder himself in 1977. Searching for the optimal RSI lengths may be the direction of further work, but it should be remembered that such an operation is endangered by overfitting. An example of RSI calculated with the above assumptions is shown in Figure 2.

### 3.2. Selection of the Data Range

The date range examined in the experiments starts on 1 January 2018 and ends on 1 January 2022, at a daily interval. The RSI values were also calculated for the 14 days before 1 January 2018. The returns were calculated until the end of March 2022. This approach allowed for the examination and evaluation of the full period between 1 January 2018 and 1 January 2022.

**Figure 3:**
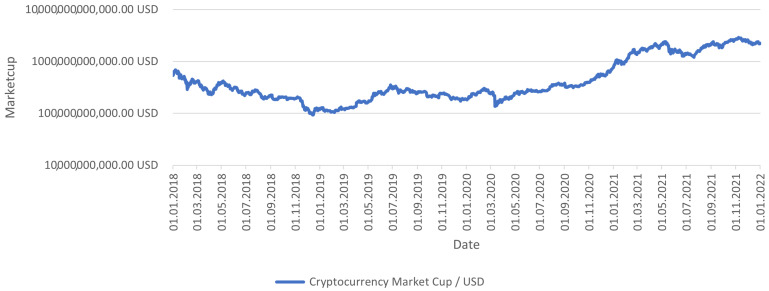
Cryptocurrency total market cup in the period under study.

The date range in which the research was carried out is equal to 4 years. The examined period was to be characterized by various market conditions (Figure 3). During the 1462 days, which constitute the research period of 4 years, several subperiods can be distinguished that are structurally significantly different from each other.

Primary research periods used to calculate the value of the RSI and the returns:

- The Bear Market Period;
- The Accumulation Period;
- The Bull Market Period;
- The Sideways Movement Period;
- The Black Swan Event;
- The Distribution Period.

The division was carried out following the best of the author’s knowledge and the available literature. The methods presented by Wycoff were used to create the division [39,40]. The division did not directly impact the research, but it shows that the analyzed period is varied. The strategies that perform well in this period can likely be pretty universal due to the need to adapt to many market conditions.

**Figure 4:**
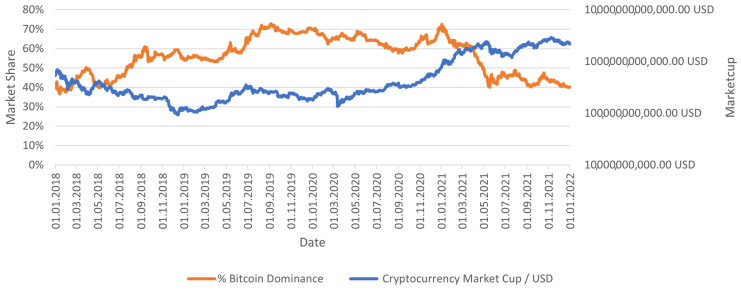
Cryptocurrency total market cup and Bitcoin dominance in the period under study.

The starting point of the research was selected due to the limited adoption of cryptocurrencies in the previous years (Figure 4); Bitcoin’s high dominance in previous years; the absence of data available for smaller projects in the previous years; the low affiliation of projects in the previous years; and the low importance of the market in the previous years.

The facts above suggest that 1 January 2018 is one of the best dates to start the research. In addition, it allows one to study a period almost equal to four years, i.e., a period equal to one Bitcoin cycle. It is not an end-to-end cycle study but a period corresponding to it, suggesting that all market behaviors typical of the bitcoin cycle should be included in the period under study. It is worth noting that with the study of the period corresponding to the full cycle, it can be assumed that regardless of where the study starts the ratio of the initial price to the final price will be relatively similar.

### 3.3. Selection of the Time Intervals

The study was conducted on a daily (1D) interval, the most popular interval in the cryptocurrency market. The interval was selected to determine the long-term, medium-term, and short-term market conditions. The interval used in the study is counted based on universal time.

The daily interval is the basic one on almost all cryptocurrency exchanges and price tracking services, including TradingView. Given the length of the RSI, the study will take into account the data from the previous 14 days (two weeks).

The weekly (1W) interval, which is probably the most popular long-term interval, and the hourly (4H) interval, which is probably the most popular hourly interval, were also considered. The results of the initial experiments on these intervals were very similar to those obtained in the daily interval. The weekly interval was characterized by more extreme results, and the four-hour interval by more moderate results. For many of the RSI applications studied, it can be assumed that the overall conclusions remain similar at 4H, 1W, and all the intervals between them.

Lower time intervals were not examined in the experiment due to problems with data availability, computational complexity, and the lower popularity of the intervals. It should also be noted that only single-interval approaches were taken into account in the experiments. The use of a multi-timeframe approach may result in higher strategy profits.

### 3.4. Selection of the Instruments

In the experiments, 11 different indexes were tested, and a portfolio was built on the basis of 10 of the selected indexes.

The most important index participating in the study was the index summarizing the value of the entire cryptocurrency market. It made it possible to examine the entire market with a single index. In the analyzed period, the value of the cryptocurrency market increased from USD536.02 billion to USD2250.75 billion, giving an increase of approximately 320%. In reality, it is impossible to invest in such an index, and most investors will achieve significantly worse returns during this period.

In order to study the most popular cryptocurrencies and cryptocurrencies with the most extreme characteristics, a sample of 10 cryptocurrencies was selected, based on the following criteria:

- Bitcoin;
- Five altcoins as of 1 January 2018 with the highest market cup;
- Altcoin most correlated with Bitcoin in the analyzed period;
- Altcoin least correlated with Bitcoin in the analyzed period;
- The most profitable altcoin in the analyzed period;
- The least profitable altcoin in the analyzed period.

Such a selection of the sample allowed the examination of the most popular and the oldest cryptocurrency, Bitcoin; the most popular altcoins; the cryptocurrencies with extreme returns in the analyzed period; and the cryptocurrencies with an extreme value of correlation with Bitcoin, which is an asset that sets the direction of the market. The sample selection was inspired by stratified sample selection.

General studies were carried out on a sample of 50 cryptocurrencies (Table 1). The data used to determine the 50 largest cryptocurrencies on the first day of the research were downloaded from the Coin Market Cup website, which allows for an overview of the market’s historical market status on the dates selected by the user [41].

To analyze the diversity, a comparison was made of the market positions of the 50 largest cryptocurrencies in the analyzed period, i.e., between 1 January 2018 and 1 January 2022. Compared at the beginning and end of the period were the market position, the change in total market capitalization, the change in dominance, and the change in market dominance.

The results of this short experiment show that when investing in altcoins, it can be challenging to achieve results that exceed the results of Bitcoin within a complete 4-year cycle (Table 1). For many investors, this fact may come as a surprise due to the high popularity of altcoins in 2021. The changes in the altcoin market in the coming years may be less drastic, but the potential risk should not be ignored. The presented results leave room for further research and make it possible to put forward many theses.

**Figure 5:**
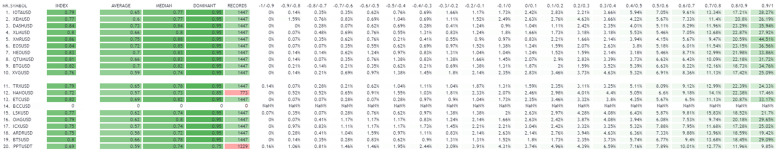
The results of the examination of the correlation between the first 20 altcoins and Bitcoin in TradingView. NaN% means no data was available. Green indicates positive results, white neutral results and red indicates that not full 1462 days of records was available.

In order to examine the correlation of the studied altcoins with Bitcoins, a program was written in the TradingView platform (Figure 5). The program allowed the examination of the correlation of a length selected by the user. In the experiment, the length of 14 was adopted due to the length of the RSI used. The correlation was studied over the 4 years of the experiment. A total of 1447 records were recorded for each index. The research was conducted daily. To examine the correlation as comprehensively as possible, a factor was created. The factor contained the mean of the average, the median, and the dominant of the collected data for each cryptocurrency. The possible range of the factor varied from −1 to 1. The experiment did not consider Bitcoin or the top five altcoins.

Every examined cryptocurrency had a relatively high correlation with Bitcoin. For every examined index, the correlation factor was higher than 0.7. For almost all the indexes, the dominance of the correlation fell within the range of 0.9–1.0.

The following altcoins were selected for the study:

- Monero (XMR)—altcoin with the highest correlation with Bitcoin—factor: 0.86; average: 0.6; median: 0.88; dominant: 0.9–1.0;
- ICON (ICX)—altcoin with the lowest correlation with Bitcoin—factor: 0.75; average: 0.57; median: 0.74; dominant: 0.9–1.0.

After conducting the research and finding the cryptocurrencies most and least correlated with Bitcoin, the cryptocurrencies with the most significant increase and the greatest decrease in price in the period studied were identified.

The most profitable cryptocurrency during the experiment was Binance Coin, a native token of the currently largest cryptocurrency exchange, Binance. During the 4 years under study, its value increased by 6003.59%. It is a project with many utilities and a dedicated team. Out of the researched projects, it was also characterized by the most significant increase in market position. On 1 January 2022, it was ranked third in market capitalization.

BitConnect (BCC) was the least profitable cryptocurrency during the experiment. As the project was a financial pyramid and it collapsed during the experiment, it was not considered in the experiment. The second most relegated project was Factom (FCT), which is no longer listed on TradingView. Another project, Salt (SALT), does not have a complete listing history available. Therefore, it was not taken into account either. For this reason, the 4th least profitable project, HyperCash (HC), was selected as the least profitable cryptocurrency. During the experiment, its price dropped by −98.75%. It is worth noting that all the projects mentioned above showed a loss between 98.75% and 100.00%. Therefore, the differences in their results should not be significant.

The final selected sample is presented as follows:

1. Bitcoin (BTC);
2. Ripple (XRP);
3. Ethereum (ETH);
4. Bitcoin Cash (BCH);
5. Cardano (ADA);
6. Litecoin (LTC);
7. Monero (XMR);
8. ICON (ICX);
9. Binance Coin (BNB);
10. HyperCash (HC).

The selected cryptocurrencies represent a diverse picture of age, market maturity, and applications. The uses of the studied cryptocurrencies range from payment systems through decentralized application platforms and NFTs to stores of value. Investors usually decide to buy significant, safe cryptocurrencies and have little exposure to more risky projects. Such a picture is presented in the studied sample. The sample allows for comprehensive market research with a relatively small number of indices without unnecessary overfitting.

### 3.5. Portfolio

In order to better evaluate the chosen sample and the results of the cryptocurrencies themselves, in most of the experiments the results of the portfolio created on their basis were additionally examined. In the conducted experiments, attention should be paid to the size and market capitalization of the cryptocurrency. The conclusions about the operation of Bitcoin should be treated as more important than the conclusions about the operation of Factom altcoin.

The composition of the developed portfolio was as follows:

1. Bitcoin (BTC)—40%;
2. Ripple (XRP)—10%;
3. Etherneum (ETH)—10%;
4. Bitcoin Cash (BCH)—10%;
5. Cardano (ADA)—10%;
6. Litecoin (LTC)—10%;
7. Monero (XMR)—2.5%;
8. ICON (ICX)—2.5%;
9. Binance Coin (BNB)—2.5%;
10. HyperCash (HC)—2.5%.

**Figure 6:**
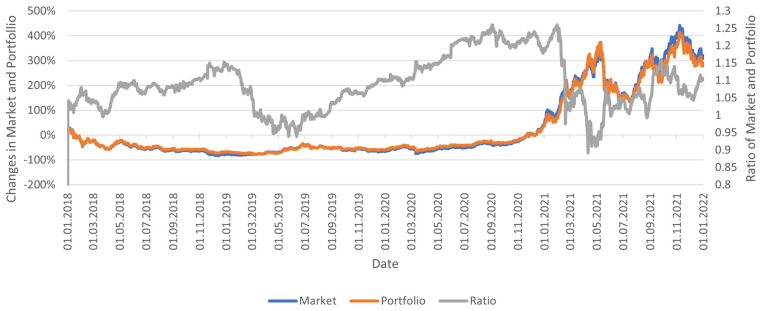
Changes in the portfolio and cryptocurrency market prices of individual analyzed indices in the analyzed period.

The composition of the accepted portfolio did not change throughout the experiment. It should be noted that this portfolio has relatively high exposure to altcoins and a relatively high percentage of capital in relatively risky indexes (10%). The wallet was developed based on the capital allocation on the market on 1 January 2022 and Bitcoin’s dominance as of 1 January 2018, amounting to 38.48. In the analyzed period, the portfolio achieved a return about 15% lower than the market, achieving a return of about 275%. It is worth noting that the constructed portfolio reflects the market quite well, although the authors’ goal was primarily to show popular and extreme cryptocurrencies that could make up a portfolio typical of an investor (Figure 6).

### 3.6. Initial Capital

No initial capital was used in the experiments. In the experiments, only the percentage gains and losses were calculated and then compared to the index under study. Thanks to the division into 100,000,000 Satoshi, Bitcoin allows the buying of any part of the entire Bitcoin with a value close to or equal to any value of the fiat currency [42]. This allows the trader to invest 100% of the portfolio capital. In the case of other cryptocurrencies, it is also possible to buy a part of a coin [43].

### 3.7. Fees and Slippage

The value of the fee was selected on the basic spot market fees of the largest cryptocurrency exchange—Binance. It is equal to 0.1% of the transaction value. It should be remembered that in most cases, the fees can be reduced with the help of referral links, the use of a native exchange token, the appropriate turnover, and the usage of appropriate stablecoins. In examining the parameters to trade on a specific exchange, it is recommended to become familiar with the exchange fee rates. The right approach can significantly help to reduce fees, thus making low timeframe trading much more profitable.

The study did not consider slippage or the impact of the simulated trader’s large orders on the market.

### 3.8. Collected Data

The analysis of returns was adopted in the study. It is the most superficial analysis of the effectiveness of the RSI as a tool for determining the reversal or continuation of the trend. The data collected during the experiment to select the optimal RSI strategies and use cases were composed primarily of returns. However, specific studies differ in the scope of the collected data. This sub-section describes the assumptions made when collecting the most critical data. The results were analyzed for the entire set of examined cryptocurrencies.

#### 3.8.1. Simple Returns

To correctly determine the value of the returns between the given candlesticks, one needs to define a relationship between the rate of return and the index price at the beginning and the end of the examined period. Simple returns or logarithmic returns can be used to describe this relationship. The experiment used simple returns due to the lower computational complexity.

The formula for a simple return is as follows:

*Simple Return* = (*Candle Close/Candle t Candles Prior*) − 1, (2)

where *t* is the number of candles.

An alternative method of analysis would be an analysis using the ZigZag indicator, which would determine not so much whether the use of the RSI is profitable, but whether the RSI correctly predicted the market movement. As an investor, however, one should primarily care about profits, which is why this approach was not used. The ZigZag approach may prove effective in predicting market movements.

The candlestick returns were examined for the RSI at the oversold area and the overbought area. The returns from these levels were then compared to the average returns of the index over the same period. The returns after 1 and 3 days were examined for short-term efficacy; after 7 and 14 days for medium-term efficacy; and after 30 and 60 days for longer-term efficacy.

#### 3.8.2. Strategy Returns

In the case of trading strategies and examining the effectiveness of the RSI in determining the trend, the strategy returns of the strategy are examined.

The formula for a strategy return is as follows:

*Strategy Return* = (*Candle Close/Candle Close at the Beginning of Trade*) × *Capital at the Beginning of Trade* (3)

The percentage of the profit obtained with the buy and hold strategy (BH) was also necessary for the analysis. In the experiment, to avoid the inconsistencies resulting from the differences between open and close, a formula was used that is identical to that used to calculate scrupulous profits. In this case, their counting condition is always proper, which allows for the obtaining of the desired property.

#### 3.8.3. Number of Appearances of the RSI Signals

The information on the number of RSI signals in the examined intervals was also collected. This information came in handy in subsequent studies. The information helped to illustrate a severe problem in the operation of the RSI. It also allowed the stating of whether the collected sample was large enough to conclude. After collecting the data, the average returns of the oversold and overbought areas were compared with the average returns of the other levels.

## 4. Results of the Experiments and Discussion

When deciding to invest in financial markets, the investor or trader tries to make the trades as good as possible. The trading strategy should minimize the risk while maximizing profits. Proper knowledge and research can allow traders to choose the best solutions.

The study’s results unambiguously made it possible to identify the applications of the RSI that may bring investors above-average results and reduce the risk resulting from following generally accepted opinions.

The wide range of the uses of the RSI was examined in order to understand its strengths and weaknesses. The precisely described scope of the examined applications is listed in Section 4.3. During the study, attention was paid to how a given strategy works for each of the analyzed indexes to examine the strategy as comprehensively as possible. The results were confronted with the generally prevailing opinions.

### 4.1. The Research Environment

For the first stage of experiments, a cryptocurrency exchange model was created in the Python environment that corresponds to the operation of an actual cryptocurrency exchange during the observed period. The model allows for any actions based on price changes and the indicated indexes’ volumes. A model of the automated trading system (ATS) was developed to obtain the results. It worked automatically by carrying out transactions when certain conditions in the field of technical analysis were met [44]. The model allowed for a quick examination of vast data ranges, which was necessary for the composite research. After the research was conducted, the best solutions for using the chosen components were examined.

For an easy and quick visualization and a more accessible selection of the studied areas, scripts were written in the TradingView Pine Script environment [45]. The scripts allowed a simulation of the operation of the stock exchange model. It should be noted that thanks to the unique structure of the Pine Script coding language used in TradingView, the exhaustive search of over 500 different returns from 50 different indexes with 1500 candles each can take less than a few seconds, which allows for a swift evaluation of the results.

In the second phase of the research, a function taken from the Pine Script libraries was used, allowing for faster calculations than with manual implementation.

### 4.2. The Research Method

The exhaustive search method was used to obtain a complete set of combinations of the results found for the strategies [46]. This method is sufficient for simple strategies, with no more than a few analyzed indexes. In the case of the advanced strategies that use more parameters, it is recommended to use a more sophisticated approach, such as evolutional algorithms, discrete optimization techniques, etc. [47]. The method of evaluating the obtained results in the case of using both techniques remains similar.

Both the simple returns on the specific RSI values after a certain period of time and the strategies for auto trading with the use of the RSI were examined. It should be noted that a large part of the strategies used with the RSI is enriched with other indicators. However, this work examines the effectiveness of the indicator solely.

It is also worth noting that the effectiveness of the strategies based on the specific signals and investments signals were treated as short, long, or sell-all position signals. This approach allowed for the greater transparency of the results.

### 4.3. Conducted Experiments

As part of the experiments, three significantly different applications of the RSI indicator will be explored. Each of them will also be assigned a trading strategy tailored to the given application.

The applications of the RSI used in the experiments are:

- Effectiveness of oversold and overbought levels in predicting trend reversals;
- Effectiveness of divergences of RSI and price in predicting trend reversals;
- Effectiveness of RSI in determining the market trend.

It is worth noting that the last application contradicts the others. It is the only examined application where the RSI is used to determine the trend. For each use, the strategies based on that use were explored. For each application, the following types of strategies were explored:

- Effectiveness of strategy taking both long and short positions;
- Effectiveness of strategy taking long positions;
- Effectiveness of strategy taking short positions;
- Buy and hold strategy: to compare the results of the examined strategies against the strategy consisting of buying an index at the beginning of the analyzed period and selling at the end of the analyzed period.

### 4.4. Effectiveness of Oversold and Overbought Levels in Predicting Trend Reversals

According to Wilder, a temporary or permanent trend reversal becomes highly likely when the RSI goes relatively too high or too low. The experiment in this section examined the effectiveness of the RSI in terms of the ability to predict local peaks and local bottoms. As part of the study, all the returns (Formula (2)) were checked in the subsequent periods when:

- The RSI indicator reached the oversold value—below 30;
- The RSI indicator reached the overbought value—above 70.

The information on the number of overbought and oversold occurrences for the given levels was collected to determine whether the examined sample was large enough.

#### 4.4.1. Results of the Experiment

The experiment showed how rapidly the cryptocurrency market was growing in 2018–2022 (Table 2). By investing in Bitcoin in this period, an investor would receive an average daily return of around 0.5% by investing when the previous RSI was either oversold or overbought. Considering the market declines in 2018, this is a fantastic investment result.

Looking at the study summary, it might seem that the most famous use of RSI is relatively effective at first glance (Table 2). For the oversold value in the short term, as many as eight cryptocurrencies were characterized by above-average returns. The high, above-average returns for the overbought level were also noticeable. After a long period after the occurrence of the RSI oversold and overbought values, there was a decrease in returns when compared to the average returns. The results can be considered relatively in line with the generally accepted opinion.

The situation changes drastically after breaking down the results into individual cryptocurrencies. In the case of Bitcoin, the disproportion between the oversold area of returns and the overbought area of returns is visible. A similar situation also applies to almost all cryptocurrencies, which increased in value over the period considered. For this reason, the authors decided to carry out an additional analysis with a breakdown of the cryptocurrencies that increased in value during the analyzed period and those that decreased in value during the analyzed period. The cryptocurrencies that increased in value in the analyzed period were marked in green.

In the case of the cryptocurrencies which increased in value during the analyzed period, after each of the analyzed periods more cryptocurrencies achieved above-average results after the RSI value hit the overbought level. These are the cryptocurrencies such as Binance Coin (BNB), Ethereum (ETH), Cardano (ADA), or Bitcoin (BTC) itself, which constitute or, with time, begin to constitute a significant part of the portfolio. In the case of the cryptocurrencies which decreased in value during the analyzed period, after each of the analyzed periods more cryptocurrencies achieved above-average results in the case of the oversold level.

Despite the initial results, which may suggest the effectiveness of the examined application of the RSI, according to the best of the authors’ knowledge it cannot be considered adequate. It has above-average effectiveness only in the case of the depreciating indexes. Such use, however, is likely to lower the gains of the cryptocurrencies that are more likely to increase in value. The investor’s goal should always be to make a profit, not to assume that the index he invests in will lose value.

#### 4.4.2. Investment Strategy

The automated trading system was built to authenticate the results of the effectiveness of the oversold and overbought levels in detecting a potential reversal trend and to show the practical application of the indicator in terms of investment and trading strategies.

The following strategies were examined:

- The strategy which enters a long position in the oversold range, i.e., 0–30, and a short position in the overbought range, i.e., 70–100;
- The strategy which enters a long position in the oversold range, i.e., 0–30;
- The strategy which enters a short position in the overbought range, i.e., 70–100;
- The buy and hold strategy: to compare the results of the examined strategies against the strategy consisting of buying an index at the beginning and selling at the end of the analyzed period.

The initial division of the portfolio changed during the experiment. The given index could invest the initial capital adjusted by all the capital it had earned or lost. After losing 100% of the assigned portfolio value, the index no longer entered any trades.

The strategy of entering long and short positions and only short positions led the portfolio to complete bankruptcy (Table 3). This is due to a 100% increase in the value of the cryptocurrencies while the strategy was on the short position.

The strategy of entering long and short positions and only short positions led the portfolio to complete bankruptcy (Table 3). This is due to a 100% increase in the value of the cryptocurrencies while the strategy was short.

The long-only strategy results of the strategy are relatively good. As many as 4 out of 10 indices made an overhold profit using the oversold RSI value as an entry signal. The returns are worse in the case of the strongly growing cryptocurrencies, which should not come as a surprise due to their strong growth in the overbought area of the RSI ranges. The entire portfolio made 177.7% profit compared to 275.22% made with buy and hold. The whole market fared even worse, reaching a profit of 20.99%. Treating the RSI oversold level as a long signal can be profitable but can involve significant losses and expose the trader to risk. The authors advise against this use.

#### 4.4.3. Summary

The experiment results show the upward, asymmetric nature of the market, which may make the primary applications of the RSI ineffective. The results also show that shorting the cryptocurrency market can be an extreme risk. Investing in oversold levels can bring an investor a significant profit but exposes his portfolio to an increase in the importance of the cryptocurrencies of a relatively declining nature.

### 4.5. Effectiveness of Divergences of RSI and Price in Predicting Trend Reversals

The RSI creator himself, Wilder, believes that divergences in the RSI and price might herald trend reversal. When the index is in an uptrend, the price hits a new local high above the previous one, the RSI value drops, and the bearish divergence is marked. According to the generally accepted opinion and the creator’s suggestion, this heralds a reversal of the bullish trend. Similarly, in the downward trend, when the price reaches a lower low and the RSI reaches a higher low, it means the appearance of a bullish divergence, which theoretically heralds a reversal of the bearish trend.

The pivotal lookback was used to search for divergence, which allows for the measuring of the relative rise and fall of the candles. One of the most popular lengths of the lookback has been adopted. The distance between the price peaks and the RSI creating divergences must be no shorter than 3 and not longer than 60 candles. This length corresponds to two months. The line used to separate the bearish and bullish divergences was set at an RSI value of 50.

In the experiment, the data were collected in the same way as in the case of the first experiment in Section 4.3 As part of the study, all the returns (Formula (2)) were checked in the subsequent periods when:

- The RSI indicator pointed to a bullish divergence value under 50;
- The RSI indicator pointed to a bearish divergence value below 50.

**Figure 7:**
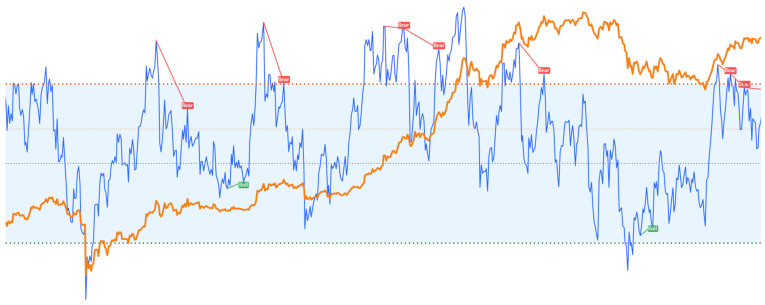
Example of the RSI Divergence, where blue stands for RSI value, orange stands for Bitcoin price, green stands for bullish divergences and red stands for bearish divergences.

An example of the occurrence of divergence on the index is shown in Figure 7. It is worth noting that the measurement of returns was made only three candles after the visible occurrences in order to avoid reference to the future.

#### 4.5.1. Results of the Experiment

The first thing that stands out in the experiment is the low number of occurrences of divergences, with an average of 0.8% of all the candles for each type of divergence. This information could mean that the signal generated by this RSI application can be considered either as not statistically significant or as a significant signal. Both hypotheses were verified by examining the strategies based on divergences.

The results alone do not conclusively demonstrate the validity or non-use of divergence signals in making investment decisions (Table 4). It is worth noting that after bearish divergences, the index usually achieved above-average results for profitable and lossy cryptocurrencies. The strategy proved most effective with Ripple (XRP), possibly due to its nature, with a lot of accumulation and distribution. The authors advise against using this strategy, especially in the case of rising cryptocurrencies such as Bitcoin (BTC) or Ethereum (ETH), where the signal works counterproductively. In the case of Bitcoin, within 60 days of the bullish divergence, the return on investment was as much as 10 times higher than in the case of the bearish divergence.

#### 4.5.2. Investment Strategy

An automated trading system was built to authenticate the results of the effectiveness of the bearish and bullish divergences in detecting a potential trend reversal and to show the practical application of the indicator in terms of the investment and trading strategies. The following strategies were examined:

- The strategy which enters a long position after bullish divergence and a short position after bearish divergence;
- The strategy which enters a long position after bullish divergence;
- The strategy which enters a short position after bearish divergence;
- The buy and hold strategy.

The original distribution of the portfolio underwent changes throughout the experiment. The specified index was able to invest the original capital based on the total capital gained or lost. Once the index lost 100% of the allocated portfolio value, it stopped making any trades.

The strategy of entering long and short positions and only short positions led the portfolio to complete bankruptcy (Table 5). This was due to a 100% increase in the value of the cryptocurrencies while the strategy was short.

In the case of the long-only strategy, a profit of 86.15% was achieved, which is about 32% of the profit achieved with the buy and hold strategy. The whole market fared slightly better, reaching a profit of 196.13%. The results were relatively negative in the case of Bitcoin (BTC) and the other profitable cryptocurrencies. The only cryptocurrency achieving spectacular profits was Ripple (XRP). This cryptocurrency achieved a profit of as much as 1074% thanks to the strategy, compared to a loss of 57% achieved with the help of buy and hold. Again, Ripple (XRP) is the cryptocurrency responsible for most of the portfolio’s profits and ends up being its main component. It reflects negatively on the strategy, the effectiveness of which is very unevenly distributed. The authors strongly advise against the use of this strategy.

#### 4.5.3. Summary

Using the RSI divergences as signals for cryptocurrency investment has proven ineffective. The experiment results and the conclusions drawn turned out to be similar to the results obtained in the first experiment. The use of the RSI divergence to predict trend reversals carries a high risk, and it is effective almost only in the case of the cryptocurrencies characterized by low-value increases over the period under review. It is possible that the automated trading system built with the help of this tool would perform even worse than the system from the Section 2. It was the most complex, the hardest to implement, and the least effective of the experiments performed.

### 4.6. RSI Efficiency in Determining the Market Trend

According to Cardwell [48], the index is in an uptrend when the values are assumed to be between 40 and 80 and in a downward trend when the values are assumed to be between 0 and 20. Due to the difficulty of determining automatically whether the values in the range of 40–60 are part of an upward or a downward trend, the values in this range were marked as having no trend. It can be argued that the following study takes an entirely different approach to the RSI, changing its nature from leading indicator to lagging indicator.

As part of the study, the sum of simple returns (Formula (2)) was examined and calculated when the previous RSI value was in the:

- Uptrend area, i.e., between 20 and 40;
- Downtrend area, i.e., between 60 and 80.

Previous experiments have shown that the primary use of the RSI for cryptocurrencies can often work in an opposite manner to the general assumptions. It can be argued that the opposite approach is likely to be effective.

#### 4.6.1. Results of the Experiment

At the beginning of the interpretation of the results of the third experiment, it is worth paying attention to a large number of occurrences of both intervals. In the case of the upward cryptocurrencies, as much as 20% of the candles had an RSI between 20 and 40. In the case of the declining cryptocurrencies, it was as much as 36 percent. Both the cryptocurrency groups had about 20% of the RSI candles in an uptrend (Table 6).

The experiment results are positive and mostly in line with Cardwell’s interpretation of the RSI (Table 6). After each analyzed period from the occurrence of the RSI-based uptrend, most of the cryptocurrencies achieved above-average results. For any period studied, no more than half of the cryptocurrencies achieved above-average results.

The cryptocurrencies that increased in value in the period under review after every period except 1 day achieved above-average results. Particularly favorable results were obtained by Bitcoin (BTC) and Cardano (ADA).

The cryptocurrencies that have decreased in value have less clearcut results. Among many of these cryptocurrencies, the performance in a downtrend was higher than in an uptrend. These are the cryptocurrencies with the highest scores in previous experiments, such as Ripple (XRP).

The results can be described as moderately positive. So, there is a chance for high results with the strategy taking long positions and not going bankrupt in the case of the strategy taking short positions.

#### 4.6.2. Investment Strategy

To authenticate the results of the effectiveness of the RSI bullish and the RSI bearish ranges in detecting trends and to show the practical application of the indicator in terms of trading strategies, an automated trading system was built. The tool used to conduct the experiment is indicated in the Supplementary Materials.

The following strategies were examined:

- The strategy which enters a long position in the uptrend range, i.e., 60–80, and a short position in the downtrend range, i.e., 20–40;
- The strategy which enters a long position in the uptrend range, i.e., 60–80;
- The strategy which enters a short position in the downtrend range, i.e., 20–40;
- The buy and hold strategy: to compare the results of the examined strategies against the strategy consisting of buying an index at the beginning of the analyzed period and selling at the end of the analyzed period.

The initial division of the portfolio changed during the experiment. The given index could invest initial capital adjusted by all the capital it had earned or lost. After losing 100% of the assigned portfolio value, the index no longer entered any trades.

Almost unambiguously positive results characterize this strategy compared to the two previous experiments (Table 7). This is the first experiment where both the long and the short strategies did not fail for all of the cryptocurrencies. The portfolio achieved a profit of 240.07% and a market 215.20%. It is quite a good result, considering the portfolio return of 275.22%. Six out of the ten cryptocurrencies surveyed achieved above-average returns. It is worth noting that HyperCash (HC) achieved a profit of over 200% instead of a loss of 98%.

The long strategy took profits of 329.24%, yielding about 20% more than buy and hold. Much better results were achieved by the entire market, by as much as 492.90%. As many as 7 out of 10 cryptocurrencies achieved above-average profits. Two out of three of the remaining cryptocurrencies made a loss which was 2 percentage points higher than buy and hold, which is a minimal difference. The only cryptocurrency with much lower yields is Binance Coin (BNB), which also rose significantly in the rest of the RSI ranges. Although the strategy primarily works against the previous ones, Ripple (XRP) also had above-average performance. After additional examination, it also turned out that the declines of the strategy relative to the entry point did not exceed 30%, which may mean that the strategy has the potential to play on leverage.

In the case of the strategy entering short positions, above-average profits were achieved by three cryptocurrencies. Most of the cryptocurrencies achieved moderate losses, around 20%. The entire portfolio achieved a loss of 19.1% and a market of −46.84% The experiment again confirms the risk associated with shorting such a volatile market while not achieving unambiguously bad results.

#### 4.6.3. Summary

The results of the strategy meet the author’s expectations, meeting all the requirements of an effective technical analysis strategy. The strategy is profitable, reduces risk, increases profit, is not overfitted, and is universal. The presented approach turned out to be safe and highly effective. In the long term, regardless of the starting point, the strategy has a great chance of exceeding the results of the examined portfolio. The results confirmed the suspicions made after the first two studies. In the case of cryptocurrencies, the RSI should be used not to look for trend reversals but rather to determine and follow the trend itself.

### 4.7. Overal Summary of the Research

The results of individual studies confirmed the author’s concerns about the ineffectiveness of the most popular adaptations of the RSI index. Contrary to the prevailing opinion, both the results of the RSI oversold/overbought levels and the divergences proved ineffective. The alternative study use of the RSI, developed by Cardwell, produced very positive and evenly distributed results.

Using the RSI to determine the trend, the last application examined was the only strategy with unambiguously positive results. With a few tweaks to the strategy, such as adding stop losses, the strategy could prove to be a simple and safe way to make consistent, moderate profits in the cryptocurrency market. The strategy significantly reduces the risk and may potentially achieve above-average results in the long term.

It is worth noting the poor results of all the strategies in the case of short positions. The authors discourage shorting cryptocurrencies using the RSI and express concern that using technical analysis in the case of the cryptocurrency market may be associated with huge risks.

In order to make a final evaluation of the algorithms, their results were compared to the results of an identical portfolio in the same research period, played based on three popular technical analysis strategies. Due to the poor results of the short position strategies, only the strategies that only played long positions were compared. The selected strategies were Supertrend, MACD, and SMA 200 Cross. Supertrend entered long positions when a signal suggesting an upward trend appeared; this was similar in the case of MACD. The strategy based on SMA 200 entered a position when the close of the index being studied closed above the average price of 200 days.

Unfortunately, when comparing the results of the selected strategy to the newer and more popular strategies, the results of the experiment do not show exceptional performance (Table 8). Even the SMA 200 Cross, which is a strategy that reacts with a relatively large delay, achieved higher results than the trend detection strategy using the RSI. Both MACD and Supertrend achieved results more than two times higher than the trend detection strategy using the RSI. It is also worth noting how good the results of Supertrend and MACD are for almost any cryptocurrency, achieving very satisfactory results.

This short study shows that technical analysis on an inefficient market, such as the cryptocurrency market, can contribute to achieving exceptional results. It should be noted that in the years 2021–2022 the cryptocurrency market showed the characteristics of a more efficient market, but there is a high likelihood that in the coming years, technical analysis will still be effective on it. This thesis may be confirmed especially in the case of cryptocurrencies with a smaller market share.

Due to the relatively weak results of all the tested strategies based on the RSI, the authors decided to study an inadequately documented strategy based on the combination of two examined strategies based on the RSI.

### 4.8. Modification of Cardwell’s Strategy

The trend-based strategy achieved relatively good results for practically every cryptocurrency studied; so, it may be a good basis for creating an effective strategy. Strategies such as Supertrend, MACD, or SMA 200 Cross often divide the studied index into two types of periods with a relatively equal number of candles. This means each of these strategies, approximately, takes long positions for about 50% of the time. The studied trend-based strategy using the RSI takes long positions for only about 20% of the time. Therefore, this strategy is very effective in terms of time exposed to the market.

In order to increase the strategy’s profits, it may be necessary to expand the range of the RSI perceived as being in an upward trend. According to tradimo.com, “A movement above 50 indicates that more traders are buying the asset than selling, and are driving the price up. If the RSI moves below 50, it shows that more traders are selling than buying, and are driving the price down.” [49]. Similar suggestions can also be found on the popular educational website for investors, investopedia.com: “As you can see in the following chart, during a downtrend, the RSI peaks near 50 rather than 70. This could be seen by traders as more reliably signaling bearish conditions.” [50]. A similar approach can be observed in the RSI divergence strategy, where, according to most of the research, a bearish divergence occurs only when the RSI is greater than 50. Many websites track indexes whose value is higher than 50, but the strategies based on this division are usually combined with another indicator. An almost identical strategy is used in the article “Technical trading and cryptocurrencies”. The authors took positions when, for a certain number of days, the RSI index value was above 50 [31,51].

The authors decided to conduct a study of a strategy based on dividing the RSI into an uptrend when the RSI for the previous candle reached a value above 50 and a downtrend when the RSI reached a value below 50. The strategy took only long positions and was compared to the remaining research in Table 9.

The strategy achieves clear positive results in the studied period. For 9/10 of the studied indexes, it achieves above-average results. The only index for which it performs worse is Binance Coin, but even there, the achieved profit exceeds 3000%. It is worth noting that this is the only strategy for which all indexes achieved a profit. Exceptionally positive results were achieved for the relatively mature index, Bitcoin. The profit achieved for this index exceeded the results achieved by buy and hold by three times, which may bode well for the effectiveness of this strategy in the future.

The development of the trend-based strategy using the RSI proved to be an exceptionally effective strategy, achieving results that surpass most new and popular cryptocurrency market strategies. It is worth noting that this strategy is a development of Cardwell’s strategy and is based on his approach to using the RSI. Both strategies can be used in a single algorithm, such as by using leverage in the range of 60–80 and spot orders or smaller exposure in the range of 50–60 and 80–100.

### 4.9. The Selected Strategy in the Post-Study Period

When examining the effectiveness of trading strategies, traders often make the mistake of matching the sample and the strategy too closely to the historical data, commonly referred to as “overfitting”. To avoid such errors, the authors examined a broad cross-section of the market over a period characterized by different market structures. To confirm the effectiveness of the sampling method and the evaluation of the trading strategies, an additional experiment was conducted.

As part of the following experiment, the strategy’s effectiveness based on the Cardwell approach with 50–100 RSI uptrend values was examined one year after the end of the study period. For this purpose, the results achieved by the portfolio from the previous experiments were examined, and a new portfolio was created on the same principles as the previous ones, but as of 1 January 2022. Due to the poor performance of the short-entry strategies, long-only strategies will be explored.

The experiment was carried out during a period of significant declines on the market. It is highly probable that after the market trend reverses, the examined strategy will obtain significantly lower returns than in the following experiment. It is worth remembering, however, that in the perspective of 4 years, the losses that the strategy avoided during the market decline should allow for returns similar to those of buy and hold. At the same time, the results of the strategy are likely to be less volatile.

The portfolio of currencies participating in the experiments achieved a return on the investment of −65.75% in the first 12 months of 2022 (Table 10). The strategy under study performed much better, with a return of −41.40%. Despite the lack of profit, its result can be considered very positive. As many as 9 out of 10 cryptocurrencies obtained above-average returns. It is worth noting that none of the studied cryptocurrencies gained value during the period under study. The experiment shows how brutal 2022 was for cryptocurrency market participants and how saving capital from loss can be as important as generating profits.

Compared to the other strategies, the results of the new application are not as outstanding as in the case of 2018–2022. It is worth noting that in 2023, characterized almost exclusively by a downward trend, the best results were achieved by the strategies that performed relatively poorly in the period 2018–2022. This fact may suggest that the traditional RSI-based strategies may be moderately effective in a downtrend. It is worth noting, however, that in the perspective of the entire 2018–2023 period, based on the RSI 50–100 range, it achieved the best results from the examined strategies. It is also highly likely that after the market reverses, this will also be the most effective strategy. The differences in the results between the individual strategies are also relatively small. It is worth noting the very good results of the RSI 60–80 strategy, which confirms the thesis put forward by the authors about the possibility of adjusting the exposure using the strategy determining the trend using the RSI. It is worth noting that for almost every strategy, the market outperformed the portfolio.

In the second part of the experiment, the composition of the second portfolio was was built as of 1 January 2022, in accordance with the rules adopted in Section 3.4 and Section 3.5.

The cryptocurrencies selected for the experiment are as follows:

1. Bitcoin (BTC)—40%;
2. Etherneum (ETH)—10%;
3. Binance Coin (BNB)—10%;
4. Solana (SOL)—10%;
5. Cardano (ADA)—10%;
6. Ripple (XRP)—10%;
7. Vechain (VET)—2.5%;
8. Helium (HNT)—2.5%;
9. LEO Token (LEO)—2.5%;
10. Terra (LUNA)—2.5%.

The returns of the second portfolio acquired with the strategy RSI 50–100 exceeded the returns of the buy and hold strategy (Table 11). The examined strategy achieved a return of −38.15% in the analyzed period, compared to a loss of −68.06% in the case of the buy and hold strategy. Above-average returns were achieved across all of the cryptocurrencies except LEO. As many as two cryptocurrencies generated profit with the help of the strategy. The strategy managed to achieve a return of −16.50% compared to the complete collapse of the Terra (LUNA) cryptocurrency, thus saving part of the portfolio from bankruptcy. Most of the other observations regarding the examined strategies remain the same as in the case of the first portfolio, confirming the universality of the solutions. It is worth noting that for almost every strategy, the market outperformed the portfolio.

The conducted experiments confirm the effectiveness of the strategy and the methodology of the research conducted in the article. The strategy selected as a result of the research for future data achieves results significantly exceeding the results of the buy and hold strategy.

### 4.10. Directions for Future Research

The promising results obtained during the experiments show that with an exhaustive search, it is possible to examine many trading strategies based on TA. The selected best of the researched strategies allowed the portfolio to achieve above-average results, even after the end of the study. It was also so universal that the new portfolio created on 1 January 2022 also achieved above-average results. Based on the presented approach, even simple strategies based only on TA can be very profitable.

It should be remembered that the selected optimal parameters for a given strategy for a historical period do not guarantee future success. However, an assumption can be made that the correct strategy decision in recent years increases the probability that the correct operation will also continue in the future, and the optimal solutions might remain in a similar range of values for many years.

TA is only one type of analysis that can be used to predict price movements effectively. We presume that the use of macroeconomic data, the correlation of cryptocurrencies with traditional markets, and on-chain data may further improve the performance of the chosen strategy [12]. Attempts to apply the analysis of the FA and the on-chain analysis data to predict cryptocurrency price movements will be the subject of further research. The future research will examine the automatic selection of parameters for different strategies according to the market conditions.

## 5. Summary

The article presents a scientific, justified approach to selecting a portfolio and researching the effectiveness signals generated by the technical analysis indicators. The proposed solution applies to algorithmic trading and can help to predict price movements and significantly reduce the risk associated with cryptocurrency trading.

The authors have developed an innovative method of building a portfolio, which allows the examination of a broad cross-section of the market using a relatively small number of indexes. The stock market model built by the authors allowed for extensive preliminary research, which has been carried out in the TradingView program.

A broad cross-section of significantly different applications and strategies based on the use of the Relative Strength Index has been examined. The authors examined the strategies designed by the creator of the indicator, J. Welles Wilder, and the well-known RSI interpreter Andrew Cardwell. Automated trading systems were built for each of the applications. The research results allowed for the selection of an RSI-based strategy that was characterized by good results for all the examined indexes. The study indicated the rather low effectiveness of the basic, popular applications of RSI.

The authors developed a new strategy, which is a modification of Cardwell’s strategy. The strategy was developed on the basis of the available literature and research conducted in the article. The selected sub-optimal application of the RSI indicator allowed the achievement of above-average results, including in the time after the end of the study. The developed strategy can compete with the most effective strategies widely used on the market.

The results of the study showed the legitimacy of the use of technical analysis in the case of the cryptocurrency market. The results of the study indicate that the cryptocurrency market may be a highly inefficient market, with a profile similar to that of emerging markets. In subsequent studies, the authors intend to focus on the study of systems that allow for the automatic selection of the parameters of the technical analysis strategies, including the RSI.

## Figures and Tables

**Table 1:** Summation of market position changes between 1 January 2018 and 1 January 2022 for the 50 highest market cup cryptocurrencies as of 1 January 2018.

	Rank Change	Market Cup Change	Price Change
**Median**	−82.50	−62.39%	−70.34%
**Average**	−201.20	244.04%	122.26%
**Gainers**	4	13	9
**Neutral**	1	0	0
**Losers**	45	37	41
**Min**	-	−100.00%	−100.00%
**Max**	32	10,187.56%	6003.59%

**Table 2:** Average returns of the examined cryptocurrencies after a selected number of days after the signal generated by the RSI application from the first experiment. In summary, the numbers indicate the number of indexes from a specific group that achieves above-average returns. Green indicates positive results, red indicates negative results.

**INDEX**		**OVERSOLD**						**OCCURENCES**	
**Nr**	**Index**	**1 Day**	**3 Days**	**7 Days**	**14 Days**	**30 Days**	**60 Days**	**NR**	**%**
1.	BTC/USD	0.49%	0.62%	1.58%	1.33%	3.45%	2.40%	158/1462	10.81%
2.	XRP/USD	2.50%	3.55%	4.10%	11.96%	9.37%	29.56%	74/1462	5.06%
3.	ETH/USD	−0.44%	−2.36%	−2.32%	3.38%	19.24%	14.75%	140/1462	9.58%
4.	BCH/USD	0.43%	1.16%	7.16%	13.69%	29.41%	10.53%	87/1462	5.95%
5.	ADA/USD	1.27%	2.76%	5.18%	7.37%	18.49%	21.38%	133/1462	9.1%
6.	LTC/USD	1.38%	1.34%	3.26%	4.22%	7.20%	7.05%	97/1462	6.63%
7.	XMR/USD	1.96%	0.96%	2.26%	3.71%	6.05%	2.14%	76/1462	5.2%
8.	ICX/USD	0.43%	−1.77%	−4.50%	0.02%	−6.49%	−13.05%	74/1462	5.06%
9.	BNB/USD	1.49%	1.26%	1.01%	3.32%	9.13%	15.08%	134/1462	9.17%
10.	HC/USD	−0.21%	1.54%	1.35%	2.46%	0.62%	11.49%	86/1462	5.88%
	MARKET	0.38%	0.22%	1.48%	4.59%	8.86%	7.01%	62/1462	4.24%
OVER AVG RESULTS BOTH		8/10	8/10	7/10	6/10	7/10	3/10	106/1462	7.24%
OVER AVG RESULTS PROFITABLE		3/4	3/4	2/4	1/4	2/4	0/4	141/1462	9.66%
OVER AVG RESULTS LOSSY		5/6	5/6	5/6	5/6	5/6	3/6	82/1462	8.45%
**INDEX**		**OVERBOUGHT**						**OCCURENCES**	
**Nr**	**Index**	**1 Day**	**3 Days**	**7 Days**	**14 Days**	**30 Days**	**60 Days**	**NR**	**%**
1.	BTC/USD	0.57%	2.11%	4.69%	8.65%	13.99%	37.04%	158/1462	10.81%
2.	XRP/USD	1.46%	4.25%	7.98%	1.13%	7.24%	−9.17%	74/1462	5.06%
3.	ETH/USD	0.35%	1.36%	2.7%	2.46%	5.41%	7.89%	140/1462	9.58%
4.	BCH/USD	−0.62%	0.08%	−2.45%	−5.79%	−11.29%	−10.79%	87/1462	5.95%
5.	ADA/USD	0.96%	3.55%	6.69%	10.49%	22.32%	41.98%	133/1462	9.1%
6.	LTC/USD	−0.61%	−2.35%	−5.06%	−5.34%	−6.00%	6.08%	97/1462	6.63%
7.	XMR/USD	0.3%	0.52%	0.43%	1.43%	−3.17%	17.75%	76/1462	5.2%
8.	ICX/USD	0.18%	2.73%	8.36%	14.65%	17.86%	9.50%	74/1462	5.06%
9.	BNB/USD	1.66%	4.90%	11.72%	24.00%	46.40%	98.29%	134/1462	9.17%
10.	HC/USD	1.33%	4.46%	8.77%	3.28%	−2.36%	−24.73%	86/1462	5.88%
	MARKET	0.21%	1.10%	2.56%	4.84%	9.08%	31.39%	161/1462	11.01%
OVER AVG RESULTS BOTH		8/10	8/10	7/10	5/10	5/10	4/10	106/1462	7.24%
OVER AVG RESULTS PROFITABLE		4/4	4/4	4/4	3/4	3/4	3/4	141/1462	9.66%
OVER AVG RESULTS LOSSY		4/6	4/6	3/6	2/6	2/6	1/6	82/1462	8.45%

**Table 3:** The results of the strategy from the first experiment for portfolio and examined cryptocurrencies. Green indicates positive returns, and red indicates negative returns.

	INDEX	BOTH	LONG	SHORT	HOLD
1.	BTC/USD	−100.00%	42.59%	−100.00%	243.79%
2.	XRP/USD	−100.00%	1372.33%	−100.00%	−57.24%
3.	ETH/USD	−100.00%	−1.26%	−100.00%	408.03%
4.	BCH/USD	−100.00%	203.21%	−100.00%	−81.20%
5.	ADA/USD	−100.00%	21.23%	−100.00%	91.31%
6.	LTC/USD	−100.00%	−48.66%	−100.00%	−33.48%
7.	XMR/USD	−100.00%	170.22%	−100.00%	−25.24%
8.	ICX/USD	−100.00%	−94.17%	−100.00%	−76.64%
9.	BNB/USD	−100.00%	214.80%	−100.00%	5998.97%
10.	HC/USD	−100.00%	−51.86%	−100.00%	−98.60%
	PORTFOLIO	−100.00%	177.70%	−100.00%	275.22%
	MARKET	−100.00%	20.99%	−100.00%	320.14%
	OVER HOLD	0/10	4/10	0/10	-
	PROFITABLE	0/10	6/10	0/10	4/10

**Table 4:** Average returns of the examined cryptocurrencies after a selected number of days after the signal generated by the RSI application from the second experiment. In summary, the numbers indicate the number of indexes from a specific group that achieves above-average returns. Green indicates positive results, red indicates negative results.

**INDEX**		**BULLISH DIVERGENCE**						**OCCURRENCES**	
**Nr**	**Index**	**1 Day**	**3 Days**	**7 Days**	**14 Days**	**30 Days**	**60 Days**	**NR**	**%**
1.	BTC/USD	0.88%	3.6%	5.27%	4.43%	8.34%	2.78%	12/1462	0.82%
2.	XRP/USD	3.10%	2.42%	12.17%	10.44%	21.44%	16.69%	5/1462	0.34%
3.	ETH/USD	0.8%	1.27%	4.74%	9.19%	14.98%	9.58%	13/1462	0.89%
4.	BCH/USD	2.36%	5.44%	1.61%	4.97%	10.04%	12.83%	13/1462	0.89%
5.	ADA/USD	−0.11%	1.49%	1.35%	6.77%	17.27%	2.48%	11/1462	0.75%
6.	LTC/USD	2.56%	2.25%	3.57%	−0.28%	−1.52%	16.99%	14/1462	0.96%
7.	XMR/USD	−2.47%	−2.08%	−2.05%	0.28%	1.89%	6.01%	16/1462	1.09%
8.	ICX/USD	−1.86%	−1.50%	−2.26%	−2.50%	−4.48%	8.13%	15/1462	1.03%
9.	BNB/USD	1.71%	−0.21%	2.35%	0.97%	4.96%	20.33%	9/1462	0.62%
10.	HC/USD	1.32%	3.23%	2.21%	2.55%	14.69%	28.85%	14/1462	0.96%
	MARKET	−0.12%	−0.24%	4.00%	6.00%	8.56%	12.79%	10/1462	0.68%
OVER AVG RESULTS BOTH		7/10	7/10	6/10	6/10	5/10	3/10	12/1462	0.83%
OVER AVG RESULTS PROFITABLE		3/4	3/4	2/4	3/4	3/4	0/4	11/1462	0.77%
OVER AVG RESULTS LOSSY		4/6	4/6	4/6	3/6	2/6	3/6	13/1462	1.32%
**INDEX**		**BEARISH DIVERGENCE**						**OCCURRENCES**	
**Nr**	**Index**	**1 Day**	**3 Days**	**7 Days**	**14 Days**	**30 Days**	**60 Days**	**NR**	**%**
1.	BTC/USD	1.44%	2.53%	3.93%	6.47%	11.32%	29.99%	1.44%	2.53%
2.	XRP/USD	−1.83%	−2%	−5.06%	−4.09%	−9.29%	−25.22%	−1.83%	−2%
3.	ETH/USD	−0.45%	0.81%	6.72%	16.57%	18.44%	43.63%	−0.45%	0.81%
4.	BCH/USD	1.46%	6.7%	17.98%	21.48%	11.29%	44.1%	1.46%	6.7%
5.	ADA/USD	0.26%	2.71%	1.97%	4.93%	16.2%	21.6%	0.26%	2.71%
6.	LTC/USD	−0.74%	−2.68%	−1.11%	−0.31%	−3.56%	12.99%	−0.74%	−2.68%
7.	XMR/USD	1.72%	1.85%	3.07%	−1.39%	−2.99%	3.46%	1.72%	1.85%
8.	ICX/USD	2.49%	8.33%	13.31%	−1.28%	−1.66%	5.19%	2.49%	8.33%
9.	BNB/USD	−2.62%	−1.55%	4.69%	19.27%	17.17%	22.1%	−2.62%	−1.55%
10.	HC/USD	1.31%	0.15%	−0.94%	4.66%	−2.35%	−23.75%	1.31%	0.15%
	MARKET	1.73%	1.82%	7.61%	7.16%	7.70%	20.83%	15/1462	1.03%
OVER AVG RESULTS BOTH		5/10	7/10	7/10	7/10	5/10	5/10	12/1462	0.81%
OVER AVG RESULTS PROFITABLE		1/4	3/4	4/4	4/4	3/4	2/4	15/1462	1.01%
OVER AVG RESULTS LOSSY		4/6	4/6	3/6	3/6	2/6	3/6	10/1462	1.03%

**Table 5:** The results of the strategy from the second experiment for portfolio and examined cryptocurrencies. Green indicates positive returns, and red indicates negative returns.

	INDEX	BOTH	LONG	SHORT	HOLD
1.	BTC/USD	−100.00%	−31.85%	−100.00%	243.79%
2.	XRP/USD	−100.00%	1074.51%	−100.00%	−57.24%
3.	ETH/USD	−100.00%	−59.71%	−100.00%	408.03%
4.	BCH/USD	−100.00%	−75.93%	−100.00%	−81.20%
5.	ADA/USD	−100.00%	104.97%	−100.00%	91.31%
6.	LTC/USD	−100.00%	−46.48%	−100.00%	−33.48%
7.	XMR/USD	−100.00%	54.07%	−100.00%	−25.24%
8.	ICX/USD	−100.00%	−14.11%	−100.00%	−76.64%
9.	BNB/USD	−100.00%	6.36%	−100.00%	5998.97%
10.	HC/USD	−100.00%	−80.12%	−100.00%	−98.60%
	PORTFOLIO	−100.00%	86.15%	−100.00%	275.22%
	MARKET	−100.00%	196.13%	−100.00%	320.14%
	OVER HOLD	0/10	6/10	0/10	-
	PROFITABLE	0/10	4/10	0/10	4/10

**Table 6:** Average returns of the examined cryptocurrencies after a selected number of days after the signal generated by the RSI application from the third experiment. In summary, the numbers indicate the number of indexes from a specific group that achieves above-average returns. Green indicates positive results, red indicates negative results.

**INDEX**		**BULLISH TREND**						**OCCURRENCES**	
**Nr**	**Index**	**1 Day**	**3 Days**	**7 Days**	**14 Days**	**30 Days**	**60 Days**	**NR**	**%**
1.	BTC/USD	0.63%	1.72%	3.51%	6.31%	10.95%	22.85%	351/1462	24.01%
2.	XRP/USD	0.18%	2.41%	6.15%	10.75%	8.69%	16.89%	210/1462	14.36%
3.	ETH/USD	0.55%	1.31%	2.72%	6.79%	11.50%	24.15%	359/1462	24.56%
4.	BCH/USD	0.99%	2.78%	4.80%	4.29%	3.36%	0.92%	264/1462	18.06%
5.	ADA/USD	0.88%	3.00%	6.49%	11.64%	25.98%	42.43%	302/1462	20.66%
6.	LTC/USD	0.06%	0.19%	0.66%	2.68%	5.15%	17.26%	287/1462	19.63%
7.	XMR/USD	0.18%	0.84%	1.09%	0.92%	−1.69%	−0.59%	287/1462	19.63%
8.	ICX/USD	0.12%	0.75%	4.27%	11.31%	26.11%	30.53%	244/1462	16.69%
9.	BNB/USD	0.42%	1.76%	4.94%	12.09%	28.62%	70.81%	367/1462	25.1%
10.	HC/USD	0.62%	3.50%	5.98%	5.16%	1.86%	−15.76%	204/1462	13.95%
	MARKET	0.49%	1.67%	3.61%	5.92%	9.67%	22.78%	412/1462	28.18%
OVER AVG RESULTS BOTH		6/10	9/10	9/10	9/10	9/10	6/10	288/1462	19.66%
OVER AVG RESULTS PROFITABLE		3/4	4/4	4/4	4/4	4/4	4/4	345/1462	23.58%
OVER AVG RESULTS LOSSY		3/6	5/6	5/6	5/6	5/6	2/6	249/1462	25.58%
**INDEX**		**BEARISH TREND**						**OCCURRENCES**	
**Nr**	**Index**	**1 Day**	**3 Days**	**7 Days**	**14 Days**	**30 Days**	**60 Days**	**NR**	**%**
1.	BTC/USD	0.13%	0.52%	1.35%	2.29%	3.50%	5.91%	271/1462	18.54%
2.	XRP/USD	0.30%	0.53%	1.76%	5.90%	9.82%	17.57%	358/1462	24.49%
3.	ETH/USD	0.03%	0.06%	−0.06%	2.07%	6.47%	12.53%	283/1462	19.36%
4.	BCH/USD	0.31%	0.42%	0.50%	1.10%	7.21%	9.05%	355/1462	24.28%
5.	ADA/USD	−0.05%	−0.34%	−0.10%	−0.92%	2.28%	6.38%	362/1462	24.76%
6.	LTC/USD	−0.1%	−0.04%	0.19%	0.54%	0.92%	1.50%	350/1462	23.94%
7.	XMR/USD	0.53%	0.19%	1.19%	4.95%	9.4%	2.86%	252/1462	17.24%
8.	ICX/USD	0.52%	0.71%	2.10%	2.02%	−1.99%	6.42%	343/1462	23.46%
9.	BNB/USD	0.48%	1.03%	2.24%	4.84%	5.48%	19.39%	215/1462	14.71%
10.	HC/USD	−0.16%	−0.21%	−0.15%	−1.31%	0.49%	10.81%	463/1462	31.67%
	MARKET	0.30%	0.47%	1.47%	3.68%	6.26%	7.43%	245/1462	16.76%
OVER AVG RESULTS BOTH		5/10	4/10	5/10	2/10	4/10	2/10	325/1462	22.24%
OVER AVG RESULTS PROFITABLE		1/4	1/4	1/4	0/4	0/4	0/4	283/1462	19.34%
OVER AVG RESULTS LOSSY		4/6	3/6	4/6	2/6	4/6	2/6	354/1462	36.27%

**Table 7:** The results of the strategy from the third experiment for portfolio and examined cryptocurrencies. Green indicates positive returns, and red indicates negative returns. In summary, “Over Hold” indicates the number of indexes that achieve above-average returns.

	INDEX	BOTH	LONG	SHORT	HOLD
1.	BTC/USD	351.55%	478.74%	−21.98%	243.79%
2.	XRP/USD	−77.28%	−39.33%	−62.55%	−57.24%
3.	ETH/USD	378.87%	462.10%	−14.81%	408.03%
4.	BCH/USD	132.32%	531.35%	−71.39%	−81.20%
5.	ADA/USD	526.72%	393.85%	26.90%	91.31%
6.	LTC/USD	9.52%	−35.07%	68.67%	−33.48%
7.	XMR/USD	−83.36%	−30.44%	−76.08%	−25.24%
8.	ICX/USD	−76.07%	18.29%	−79.77%	−76.64%
9.	BNB/USD	40.52%	244.36%	−59.19%	5998.97%
10.	HC/USD	216.37%	25.84%	15.48%	−98.60%
	PORTFOLIO	240.07%	329.24%	−19.10%	275.22%
	MARKET	215.20%	492.90%	−46.84%	320.14%
	OVER HOLD	6/10	7/10	3/10	-
	PROFITABLE	7/10	7/10	3/10	4/10

**Table 8:** The results of strategies from experiments compared to popular trading strategies between 2018 and 2022. Green indicates positive results, red indicates negative results.

	TYPE	TA			RSI			HOLD
	STRATEGY	SUPERTREND	MACD	SMA 200	RSI 60–80	DIVERGENCE	OVERSOLD	HOLD
1.	BTC/USD	500.85%	408.84%	28.26%	478.74%	−31.85%	42.59%	243.79%
2.	XRP/USD	−63.13%	1161.26%	−86.32%	−39.33%	1074.51%	1372.33%	−57.24%
3.	ETH/USD	736.84%	572.72%	423.45%	462.10%	−59.71%	−1.26%	408.03%
4.	BCH/USD	123.41%	242.31%	−29.70%	531.35%	−75.93%	203.21%	−81.20%
5.	ADA/USD	1657.05%	634.80%	2669.11%	393.85%	104.97%	21.23%	91.31%
6.	LTC/USD	220.06%	60.23%	−42.83%	−35.07%	−46.48%	−48.66%	−33.48%
7.	XMR/USD	129.40%	168.86%	1.74%	−30.44%	−54.07%	170.22%	−25.24%
8.	ICX/USD	−36.61%	−20.50%	−47.68%	18.29%	−14.11%	−94.17%	−76.64%
9.	BNB/USD	7605.38%	4190.80%	2503.31%	244.36%	6.36%	214.80%	5998.97%
10.	HC/USD	124.76%	20.04%	−84.85%	25.84%	−80.12%	−51.86%	−98.60%
	PORTFOLIO	663.34%	539.65%	363.99%	329.24%	86.15%	177.70%	275.22%
	MARKET	585.35%	833.42%	318.45%	492.90%	196.13%	20.99%	320.14%
	OVER HOLD	9/10	9/10	6/10	7/10	6/10	4/10	-
	PROFITABLE	8/10	9/10	5/10	7/10	3/10	6/10	4/10

**Table 9:** The results of strategies from experiments compared to popular trading strategies and strategy proposed by authors between 2018 and 2022. Green indicates positive results, red indicates negative results.

TYPE	TA			RSI				HOLD
STRATEGY	SUPERTREND	MACD	SMA 200	RSI 50–100	RSI 60–80	DIVERGENCE	OVERSOLD	HOLD
BTC/USD	500.85%	408.84%	28.26%	723.78%	478.74%	−31.85%	42.59%	243.79%
XRP/USD	−63.13%	1161.26%	−86.32%	178.70%	−39.33%	1074.51%	1372.33%	−57.24%
ETH/USD	736.84%	572.72%	423.45%	1159.62%	462.10%	−59.71%	−1.26%	408.03%
BCH/USD	123.41%	242.31%	−29.70%	147.06%	531.35%	−75.93%	203.21%	−81.20%
ADA/USD	1657.05%	634.80%	2669.11%	1897.46%	393.85%	104.97%	21.23%	91.31%
LTC/USD	220.06%	60.23%	−42.83%	538.72%	−35.07%	−46.48%	−48.66%	−33.48%
XMR/USD	129.40%	168.86%	1.74%	98.76%	−30.44%	−54.07%	170.22%	−25.24%
ICX/USD	−36.61%	−20.50%	−47.68%	218.31%	18.29%	−14.11%	−94.17%	−76.64%
BNB/USD	7605.38%	4190.80%	2503.31%	3321.91%	244.36%	6.36%	214.80%	5998.97%
HC/USD	124.76%	20.04%	−84.85%	40.40%	25.84%	−80.12%	−51.86%	−98.60%
PORTFOLIO	663.34%	539.65%	363.99%	773.65%	329.24%	86.15%	177.70%	275.22%
MARKET	585.35%	833.42%	318.45%	1161.21%	492.90%	196.13%	20.99%	320.14%
OVER HOLD	9/10	9/10	6/10	9/10	7/10	6/10	4/10	-
PROFITABLE	10/10	9/10	5/10	10/10	7/10	3/10	6/10	4/10

**Table 10:** The results of strategies from experiments compared to popular trading strategies and strategy proposed by authors between 2022 and 2023. Green indicates positive results, red indicates negative results.

TYPE	TA			RSI				HOLD
STRATEGY	SUPERTREND	MACD	SMA 200	RSI 50–100	RSI 60–80	DIVERGENCE	OVERSOLD	HOLD
BTC/USD	−43.06%	−39.85%	0.00%	−42.43%	−20.91%	−46.62%	42.51%	−64.04%
XRP/USD	−22.46%	−26.47%	−34.32%	−41.92%	−28.90%	−4.59%	1372.09%	−59.20%
ETH/USD	−15.39%	−25.28%	−19.34%	−14.03%	−18.24%	113.04%	−1.17%	−67.36%
BCH/USD	−51.41%	−44.84%	0.00%	−45.34%	−23.20%	−69.82%	203.32%	−77.50%
ADA/USD	−68.18%	−63.19%	0.00%	−57.20%	−8.64%	−72.77%	21.49%	−80.89%
LTC/USD	−55.94%	−38.56%	−5.73%	−41.07%	−21.69%	−36.89%	−48.20%	−51.49%
XMR/USD	−40.90%	−19.86%	−55.19%	−56.95%	−43.70%	−3.59%	170.83%	−35.07%
ICX/USD	−58.06%	−70.17%	0.00%	−48.05%	−10.20%	−80.09%	−94.77%	−88.33%
BNB/USD	−22.38%	11.71%	−37.97%	−30.11%	−6.75%	−21.22%	214.22%	−52.22%
HC/USD	−43.33%	−61.85%	0.00%	−43.91%	−13.67%	−39.42%	−51.37%	−83.94%
PORTFOLIO	−42.68%	−38.12%	−8.27%	−41.40%	−19.64%	−29.36%	−34.20%	−65.75%
MARKET	−21.52%	−31.38%	−7.79%	−31.73%	−15.33%	−34.54%	−41.69%	−65.26%
OVER HOLD	8/10	10/10	9/10	9/10	9/10	10/10	10/10	-
PROFITABLE	0/10	1/10	0/10	0/10	0/10	1/10	2/10	0/10

**Table 11:** The results of strategies from experiments compared to popular trading strategies and strategy proposed by authors between 2022 and 2023 on a new portfolio. Green indicates positive results, red indicates negative results.

TYPE	TA			RSI				HOLD
STRATEGY	SUPERTREND	MACD	SMA 200	RSI 50–100	RSI 60–80	DIVERGENCE	OVERSOLD	HOLD
BTC/USD	−43.06%	−39.85%	0.00%	−42.43%	−20.91%	−46.62%	−37.51%	−64.04%
ETH/USD	−15.39%	−25.28%	−19.34%	−14.03%	−18.24%	113.04%	−2.47%	−67.34%
BNB/USD	−22.38%	11.71%	−37.97%	−30.11%	−6.75%	−21.22%	−26.22%	−52.22%
SOL/USD	−40.74%	−78.51%	0.00%	−44.04%	−21.56%	−76.47%	−85.92%	−94.13%
ADA/USD	−68.18%	−63.19%	0.00%	−57.20%	−8.64%	−72.77%	−67.49%	−80.89%
XRP/USD	−22.46%	−26.47%	−34.32%	−34.93%	−28.90%	−4.59%	11.02%	−59.13%
VET/USD	−47.44%	−47.84%	0.00%	−5.76%	12.19%	−58.82%	7.02%	−80.61%
HNT/USD	−76.68%	−86.00%	−55.71%	−69.54%	−20.61%	−92.45%	−93.25%	−96.00%
LEO/USD	−26.31%	3.66%	19.30%	−40.24%	24.08%	−11.81%	0.00%	−6.39%
LUNA/USD	−51.04%	−28.02%	−6.39%	−16.50%	−18.21%	−100.00%	−100.00%	−100.00%
PORTFOLIO	−39.18%	−38.07%	−10.23%	−38.15%	−16.84%	−31.42%	−34.20%	−68.06%
MARKET	−21.52%	−31.38%	−7.79%	−31.73%	−15.33%	−46.62%	−41.69%	−65.26%
OVER HOLD	8/10	10/10	9/10	9/10	9/10	8/10	9/10	-
PROFITABLE	0/10	2/10	1/10	0/10	2/10	1/10	2/10	0/10

## Data Availability

The data concerning indexes has been taken from the service Trading View (https://www.tradingview.com/, accessed on 24 December 2022).

## Supplementary Materials

The trading strategy described in Section 4.6 presented on the TradingView platform: https://www.tradingview.com/script/Fm9EaxQx-MAREK-ZATWARNICKI-ARTICLE-RSI-TRADING/ (accessed on 24 December 2022).
